# Zika Virus infection and microcephaly: anxiety burden for women

**DOI:** 10.11604/pamj.2018.30.2.11794

**Published:** 2018-05-03

**Authors:** Ikenna Desmond Ebuenyi, Soumitra Sudip Bhuyan, Luchuo Engelbert Bain

**Affiliations:** 1 Athena Institute, Faculty of Earth and Life Sciences, Vrije Universiteit, Amsterdam, Netherlands; 2 Division of Health Systems, Management and Policy, University of Memphis, USA

**Keywords:** Zika virus, microcephaly, anxiety

## Abstract

The re-emergence of Zika virus in Brazil and other contiguous countries is a source of anxiety for pregnant women on account of its association with microcephaly. Adverse pregnancy outcome has huge mental health implications. It is essential for health providers to incorporate psychosocial care as part of pre and postnatal care for women in all countries affected by the Zika virus infection.

## Commentary

The resurgence of Zika virus in Brazil and the dire medical implications for the populace and pregnant women, in particular, led to its declaration as a public health emergency in November 2015 [1]. This declaration was in response to the widespread association of Zika virus with microcephaly, abortions, and deaths. The fears and concern of the Brazilian health authorities were confirmed as about some months later; the World Health Organization made a similar declaration amidst growing global concerns about Zika virus. As at November 2016, evidence of Zika virus infection (ZIKV) had been reported in over 67 countries and territories since the first report in 2015 [1]. In the light of new evidence for a causal link between Zika virus infection and microcephaly, there is heightened uncertainty and anxiety for women of reproductive age in Brazil and all over the world who are faced with the dilemma of a turning off their biological clock in the face of fatal consequences of microcephaly. The association of Zika virus with pregnancy and microcephaly is perhaps its greatest threat [1]. Pregnancy is an important event in the lives of women and a time of uncertainty. Pregnant women and the fetus are vulnerable to infectious disease, and history is fraught with examples of the effect of infectious disease on pregnancy [2, 3]. Susceptibility to infectious diseases is heightened in pregnancy with associated negative outcomes for both the fetus and women [2, 3]. Zika virus infection is a threat to the reproductive rights of women. The threat to the fetus and possibility of microcephaly is a burden and source of anxiety which has huge mental health implications [4]. Women in affected countries have to live with the fear of abortions due to Zika virus infection and the inability to procure abortion following a prenatal diagnosis of ZIKV [5]. Studies are in agreement that pregnancy is a source of worry for pregnant women and indeed the startling statistics of child death, and microcephaly would be a source of trauma for both mothers who have suffered a loss and those who are still pregnant [2, 3]. The true extent of the psychological trauma faced by the affected women is unknown but like in all epidemics as shown in the recent Ebola outbreak, a burdensome atmosphere of fear, anxiety, and uncertainty pervades in the local communities and indeed contiguous communities in the whole of Latin America [2, 6, 7]. The chronic effect of maternal psychosocial stress on maternal health and wellbeing has been demonstrated and hence the need to take proactive steps to salvage the health of women in Brazil and other affected areas. What should be done in the light of this uncertainty and fatal associations between Zika Virus and fatal pregnancy outcomes?

### Recommendations

The WHO and the Brazilian health authorities must be commended for recognizing and declaring the Zika virus outbreak a public health emergency and forming a multidisciplinary committee but so much needs to be done and lessons drawn from the recent Ebola outbreak. Ultimately, all-inclusive health systems approach that combines flexibility with effectiveness is needed to achieve the health objectives. A three-pronged approach is recommended:

**Strengthening of the health facilities in Brazil and other neighboring countries on emergency preparedness and proactive handling of the Zika virus through adequate testing, surveillance and case management:** There should be an aggressive contact tracing and provision of requisite equipment for laboratory testing and diagnosis of all suspected cases of Zika Virus. Psychosocial support is very essential and the long term effects of anxiety and trauma (post-traumatic stress disorder (PTSD) must be expected in the women that lost their pregnancy or babies during the outbreak. Psychotherapy for pregnant women and families affected is essential. Clinical psychologist and psychiatrist should be included in the high-level team set up by affected countries to address the health challenges. The WHO guideline on the provision of psychosocial support for women and families affected by Zika virus [8] should be adopted by health care providers in all countries affected by ZIKV.

**Community engagement and participation by all stakeholders:** Productive dialogue and information channels must be created to serve as a two-way information link between the communities, women the health authorities. Community engagement would foster understanding and dispel the atmosphere of misinformation and fear that subsist in similar epidemics [6]. Aggressive public health information in the affected communities would ameliorate the fear and uncertainty being experienced by women and help people make informed decisions.

**Adequate prevention and Vaccine development of a vaccine is essential:** The proactive prevention guidelines set up by the Public Health England (PHE) and the Royal College of Obstetrics and Gynaecology is recommended for health care providers everywhere [7]. Zika virus has been around for quite some time and perhaps neglected because of the mild presentations [9-11]. Time has shown that every infectious disease has the potential to be lethal and perhaps the best remedy is a vaccine. Vaccine development involves time and finance but also government, and international donors should commit to the world free from the ill effects of infectious disease. It is pertinent to state that region-specific models are indispensable in managing emergency epidemics like Zika virus outbreaks. Conflicts between traditionally held beliefs and conceptions about disease might clash with classical evidence-based management plans affecting uptake of life-saving interventions [12].

## Conclusion

Zika virus is major public health issue with implications for women of reproductive age. From the health systems perspective, it is important to adequately allocate resources, build capacity, and develop appropriate clinical protocols for the healthcare workforce for diagnosis and treatment. Emergency response preparedness in virus outbreaks is glaringly sub-optimal and the mental health needs of affected individuals must be made a priority.
